# Increased investment in gametocytes in asymptomatic *Plasmodium falciparum* infections in the wet season

**DOI:** 10.1186/s12879-020-05761-6

**Published:** 2021-01-09

**Authors:** Colins O. Oduma, Sidney Ogolla, Harrysone Atieli, Bartholomew N. Ondigo, Ming-Chieh Lee, Andrew K. Githeko, Arlene E. Dent, James W. Kazura, Guiyun Yan, Cristian Koepfli

**Affiliations:** 1 Department of Biochemistry and Molecular Biology, Egerton University, P. O Box 536, Nakuru, 20115 Kenya; 2 Kenya Medical Research Institute/Centre for Global Health Research, P. O Box 1578, Kisumu, 40100 Kenya; 3 School of Public Health, Maseno University, P. O Box 3275, Maseno, 40100 Kenya; 4 International Center of Excellence for Malaria Research, P. O Box 199, Homa Bay, 40300 Kenya; 5 Laboratory of Malaria Immunology and Vaccinology, National Institute of Allergy and Infectious Diseases, National Institute Health, Bethesda, MD 20892 USA; 6 Program in Public Health, College of Health Sciences, University of California, Irvine, CA 92697 USA; 7 Case Western Reserve University, Center for Global Health and Diseases, LC 4983, Cleveland, OH 44106 USA; 8 Eck Institute for Global Health and Department of Biological Sciences, University of Notre Dame, Notre Dame, IN 46556-0369 USA

**Keywords:** *Plasmodium falciparum*, Transmission, Gametocyte, Asymptomatic, Season

## Abstract

**Background:**

Transmission stemming from asymptomatic infections is increasingly being recognized as a threat to malaria elimination. In many regions, malaria transmission is seasonal. It is not well understood whether *Plasmodium falciparum* modulates its investment in transmission to coincide with seasonal vector abundance.

**Methods:**

We sampled 1116 asymptomatic individuals in the wet season, when vectors are abundant, and 1743 in the dry season, in two sites in western Kenya, representing different transmission intensities (Chulaimbo, moderate transmission, and Homa Bay, low transmission). Blood samples were screened for *P. falciparum* by qPCR, and gametocytes by *pfs25* RT-qPCR.

**Results:**

Parasite prevalence by qPCR was 27.1% (Chulaimbo, dry), 48.2% (Chulaimbo, wet), 9.4% (Homabay, dry), and 7.8% (Homabay, wet). Mean parasite densities did not differ between seasons (*P* = 0.562). *pfs25* transcripts were detected in 119/456 (26.1%) of infections. In the wet season, fewer infections harbored detectable gametocytes (22.3% vs. 33.8%, *P* = 0.009), but densities were 3-fold higher (wet: 3.46 transcripts/uL, dry: 1.05 transcripts/uL, *P* < 0.001). In the dry season, 4.0% of infections carried gametocytes at moderate-to-high densities likely infective (> 1 gametocyte per 2 uL blood), compared to 7.9% in the wet season. Children aged 5–15 years harbored 76.7% of infections with gametocytes at moderate-to-high densities.

**Conclusions:**

Parasites increase their investment in transmission in the wet season, reflected by higher gametocyte densities. Despite increased gametocyte densities, parasite density remained similar across seasons and were often below the limit of detection of microscopy or rapid diagnostic test, thus a large proportion of infective infections would escape population screening in the wet season. Seasonal changes of gametocytemia in asymptomatic infections need to be considered when designing malaria control measures.

**Supplementary Information:**

The online version contains supplementary material available at 10.1186/s12879-020-05761-6.

## Background

Malaria transmission stemming from asymptomatic individuals has gained attention as an increasing number of countries aims for malaria elimination rather than control. Most tools for intervention, such as bed nets, or indoor residual spraying (IRS), were developed and tested to reduce the number of clinical cases [1]. Their impact on asymptomatic infections and their transmission potential is little understood. Approaches to specifically identify and treat asymptomatic infections, such as reactive case detection [2], mass drug administration [3], or combinations thereof [4] are increasingly trialed or implemented. A better understanding of how to best use them to minimize transmission from asymptomatic carriers is needed.

In many settings with pronounced seasonality in rainfall, *Anopheles* mosquitoes are sparse in the dry season as opposed to wet season where they are plentiful, resulting in transmission primarily occurring during and shortly after the wet season [5–9]. It is not known how far *P. falciparum* adapts its transmission potential to changes in vector abundance across seasons. Adaptions to increase transmission potential when chances for onward transmission are high could maximize the fitness of the parasite population. Understanding such adaptations are crucial when introducing transmission-reducing interventions.

Over the course of the red blood cell cycle, a small proportion of *P. falciparum* parasites develop into gametocytes, the sexual form of the parasite [10]. A mosquito blood meal needs to contain at least one female and one male gametocyte to be infective [11, 12]. The ingested gametocytes develop into oocysts and after approximately two weeks, into sporozoites that are transmitted to the next vertebrate host [13]. *P. falciparum* gametocytes exist in five morphologically distinguishable stages [14]. Early ring stage gametocytes circulate in peripheral blood [15] while late stages I-IV sequester for 7 to 12 days in inner organs including bone marrow and spleen until maturity [12, 16, 17]. The mature stage V gametocytes re-enter the peripheral circulation where they require an additional 3 days to become fully infective [18, 19]. Stage V gametocytes remain in the circulation for a mean period of 6.4 days to a maximum of 3 weeks [17]. Due to the sequestration of developing gametocytes, they are rarely detected in peripheral blood during the first two weeks following sporozoite inoculation.

A large proportion of all *P. falciparum* infections remain asymptomatic. Untreated infections can persist for several months [20–22]. During this time, parasite densities fluctuate and are often below the limit of detection by microscopy. Transmission stemming from asymptomatic infections is a key obstacle for malaria control and elimination. Such subpatent *P. falciparum* gametocyte carriers have the potential to infect mosquitoes [23–25], though their contribution to transmission in different settings is not known [26]. A previous study in western Kenya found asymptomatic individuals to be more infective than clinical cases [27]. Even after antimalarial treatment, gametocytes may continue to circulate for up to 2–3 weeks [28]. Gametocyte densities are an important measure to predict the infectiousness of humans to mosquitoes [23–25], and thus useful for evaluating the effects of interventions that aim to reduce transmission [26].

Gametocyte density in the blood is governed by the conversion rate, i.e., the proportion of early ring stage parasites committed to sexual vs. asexual development. Changes in the density of mature gametocyte could be achieved through different strategies, e.g. a change of the conversion rate, growing a higher density of asexual parasites before any gametocytes develop, longer circulation of mature gametocytes, or a combination of these factors [29]. In all cases, a higher density of gametocytes will increase transmission if vectors are present. On the other hand, the investment in gametocytes is lost if gametocytes are not taken up by mosquitoes. The factors affecting the conversion rate are not well understood. In laboratory culture and rodent malaria models, factors such as high parasite density [30] and drug pressure [31] have been found to impact gametocyte conversion. Few studies have measured the conversion rate directly in natural infections and observed pronounced variation [32–34].

Areas of western Kenya experience perennial malaria transmission with peaks in vector density and transmission coinciding with seasonal rains in April–August and October–November [5, 35]. It is not known whether asymptomatic *P. falciparum* infections modulate the investment in gametocytes to coincide with the appearance of vectors at the start of transmission period.

To understand seasonal changes in gametocyte carriage in sites of differential transmission intensity, we compared *P. falciparum* gametocyte densities in asymptomatic individuals between the dry and wet seasons in a low-transmission setting (Homa Bay) and a moderate-transmission setting (Chulaimbo) in western Kenya. Blood stage parasites were diagnosed by *var*ATS qPCR, and mature female gametocytes were quantified using *pfs25* reverse transcriptase qPCR.

## Methods

### Study sites and participants

**Fig. 1:**
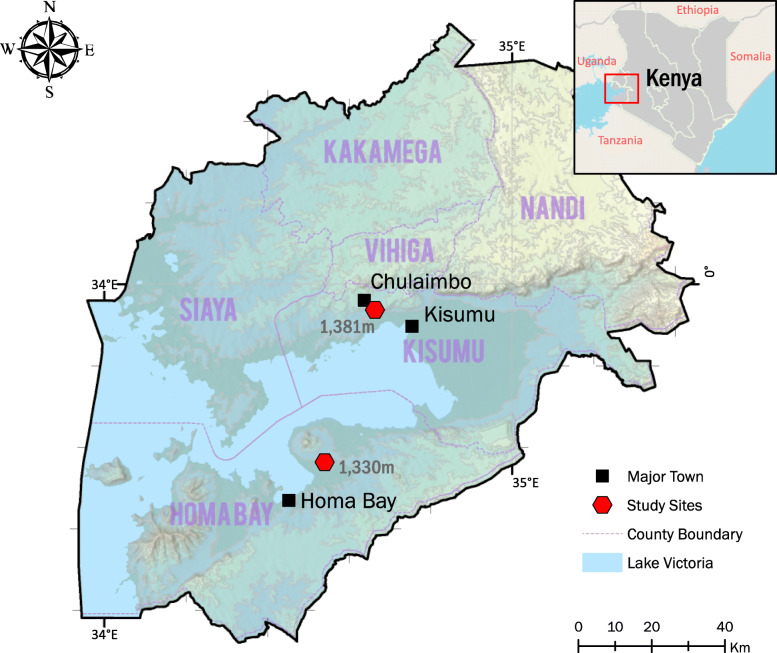
Map of study sites

2859 asymptomatic individuals were sampled in cross-sectional surveys in the dry season (*n* = 1116) between January and March 2019, and wet season (*n* = 1743) between June and August 2019 in Western Kenya, in Homa Bay (low transmission) and Chulaimbo (moderate transmission) (Table 1, Fig. 1). In these areas, *P. falciparum* is the primary malaria parasite species [36]. The study population included asymptomatic individuals aged 2 months to 99 years with no clinical symptoms. None of the study participants had been treated with antimalarial drugs within the three days prior blood sampling.

**Table 1:** Demographic and parasitological characteristics of study participants

	Chulaimbo			Homa Bay		
	dry *N* = 262	wet *N* = 419		dry *N* = 854	wet *N* = 1324	
Demographic data						
Age group in years	N (%)	N (%)		N (%)	N (%)	
< 5	45 (17.2)	62 (14.8)		158 (18.5)	227 (17.1)	
5–15	75 (28.6)	173 (41.3)		154 (18.0)	419 (31.6)	
> 15	142 (54.2)	184 (43.9)		542 (63.5)	678 (51.2)	
Female (%)	155 (59.2)	234 (55.8)		578 (67.7)	840 (63.4)	
**Parasitological data**			*P* value			*P* value
Parasite prevalence	71/262 (27.1%)	202/419 (48.2%)	< 0.0001*	80/854 (9.4%)	103/1324 (7.8%)	0.1921
Geometric mean parasite density	7.79 [3.07–19.7]	11.7 [6.80–20.2]	0.8433	6.87 [3.53–13.4]	5.31 [2.88–9.81]	0.9638
Proportion subpatent infections (< 100 parasites/μL)	48/71 (67.6%)	135/202 (66.8%)	0.9050	63/80 (78.8%)	83/103 (80.6%)	0.7595
Population gametocyte prevalence	27/262 (10.3%)	50/419 (11.9%)	0.5140	24/854 (2.8%)	18/1324 (1.4%)	0.0162*
Proportion gametocyte positive infections	27/71 (38.0%)	50/202 (24.8%)	0.0325*	24/80 (30.0%)	18/103 (17.5%)	0.0457*
Geometric mean *pfs25* density	1.37 [0.79–2.36]	4.74 [2.36–9.55]	0.0181*	0.77 [0.54–1.10]	1.42 [0.76–2.65]	0.0638

Numbers in brackets are 95% confidence intervals; asterisk (*) indicate significant at *P* < 0.05; densities are determined using T-test; and differences in prevalence using the Chi-Square test

In Chulaimbo, *Anopheles arabiensis* is the primary vector. It is abundant in the wet season. *An. gambiae s.s* is the second predominant mosquito vector [5]. In Homa Bay, *An. funestus* has re-emerged as the predominant species following development of pyrethroid resistance [37]. According to the Kenya “End of Spray” Report (2018) [38], indoor residual spraying (IRS) in Homa Bay has resulted in reduction in malaria vector densities and sporozoite rates compared with Chulaimbo where IRS has not been implemented.

### Sample collection and processing

350–400 μL of capillary blood was collected into EDTA microtainer tubes (Becton Dickinson, New Jersey, United States) by finger prick. For RNA preservation, 100 μL of whole blood was transferred to a tube containing 500 μL of RNAlater (Sigma-Aldrich, Missouri, United States) within 2 h of collection and stored at − 80 °C until RNA extraction [39, 40]. The remaining blood was centrifuged, plasma removed and stored at − 20 °C. The red cell pellet was stored at − 20 °C until DNA extraction.

### Molecular parasite screening and quantification

DNA was extracted from 100 μL blood using the Genomic DNA Extraction kit (Macherey-Nagel, *Düren,* Germany) and eluted in an equivalent volume of elution buffer. DNA was screened for *P. falciparum* using ultrasensitive qPCR that amplifies a conserved region of the *var* gene acidic terminal sequence (*var*ATS) according to a previously published protocol [41]. The *var*ATS gene assay amplifies ~ 20 copies/genome [41]. The qPCR results were converted to *var*ATS copies/μL using external standard curve of ten-fold serial dilutions (5-steps) of 3D7 *P. falciparum* parasites quantified by droplet digital PCR (ddPCR) [42]. The ddPCR thermocycling conditions, sequences and concentration of primers and probe are given in additional file 1. Asexual parasite densities were calculated by dividing *var*ATS copy numbers by 20, reflecting the approximate number of copies per genome.

For all the gametocytes assays, RNA was extracted using the pathogen Nucleic Acid Extraction kit (Macherey-Nagel, *Düren*, Germany) and eluted in 50 μL elution buffer, i.e., RNA was concentrated two-fold during extraction. RNA samples were DNase treated (Macherey-Nagel, *Düren*, Germany) to remove genomic DNA that could result in a false positive *pfs25* signal [43]. A subset of RNA samples was tested by varATS qPCR, and all tested negative.

### Molecular gametocyte screening and quantification

For gametocyte detection by quantitative reverse - transcription PCR (RT-qPCR), RNA was extracted from all *P. falciparum* qPCR-positive samples. Gametocytes were quantified by targeting female *pfs25* mRNA transcripts using one-step RT-qPCR assays (Alkali Scientific, Florida, United States). All qPCR conditions, sequences and concentration of primers and probes are given in additional file 1. The *pfs25* RT-qPCR results were converted to *pfs25* transcript copies/μL using external standard curve of ten-fold serial dilutions (5-steps) of DNA of 3D7 culture parasites quantified by ddPCR (additional file 1). Previous studies have shown that 10–20 *pfs25* transcripts are detected per gametocyte (reported as 90 transcripts/gametocyte when measured against a circular plasmid, which reflects a 5 to 10-fold overestimate) [40, 42].

### Statistical analysis

Parasite and gametocyte densities were log_10_ transformed and geometric means per μL blood calculated whenever densities were reported. The Shapiro-Wilk test and graphical normality was employed to determine normal distribution of data following log transformation. Differences in prevalence between seasons and sites were determined using the *χ*^*2*^ *test.* Differences in densities between seasons and sites were determined using T-test. Differences in densities between age-groups were determined by ANOVA’s Tukey’s multiple comparisons test. Multivariable analysis was employed to determine association of age, site and season with asexual parasite and gametocyte positivity and density. The associations were investigated by linear and logistic regression analysis. Pearson’s correlation test was conducted to establish the relationship between asexual parasite and gametocyte densities. Analysis was done in GraphPad Prism version 8 and STATA version 14.

## Results

### Prevalence and density of *P. falciparum* infections

2859 samples with age distribution representative of the population were analyzed in this study. The demographic characteristics of the study participants are summarized in Table 1.

**Fig. 2:**
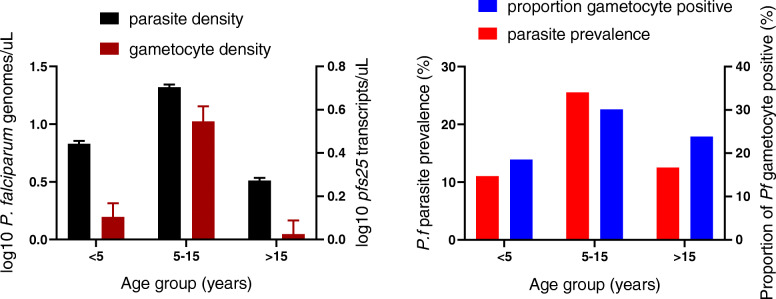
Age trends in *P. falciparum* parasite and gametocyte prevalence and density. Blood stage parasite density was measured by *var*ATS qPCR, and gametocyte density by *pfs25* mRNA RT-qPCR. The proportion of gametocyte positive infections refers to proportion of all individuals with blood stage parasite who were positive for gametocytes

Across both sites, parasite prevalence by qPCR was 13.5% in the dry season and 17.5% in the wet season (Table 2, site specific-data presented in Table 1). In both seasons, prevalence of *P. falciparum* infection was significantly higher in Chulaimbo than Homa Bay (wet: 48.2% vs. 7.8%, *P* < 0.001, dry: 27.1% vs. 9.4%, *P* < 0.001, Table 1)*.* In Chulaimbo, the prevalence was significantly higher in the wet season (*P* < 0.001, Table 1), but it did not differ between seasons in Homa Bay (*P* = 0.192, Table 1). Across all surveys, prevalence was higher in males than females (21.4% vs. 12.8%, *P* < 0.001). School-age children (5–15 years) were at highest risk of infection (Fig. 2).

**Table 2:** Parasitological characteristics of study participants by season

	dry N = 1116	wet N = 1743	
**Parasitological data**			*P* value
Parasite prevalence	151/1116 (13.5%)	305/1743 (17.5%)	0.0047*
Geometric mean parasite density	7.29 [4.18–12.7]	8.98 [5.92–13.6]	0.5620
Proportion subpatent infections (< 100 parasites/μL)	111/151 (73.5%)	218/305 (71.5%)	0.6483
Population gametocyte prevalence	51/1116 (4.6%)	68/1743 (3.9%)	0.3826
Proportion gametocyte positive infections	51/151 (33.8%)	68/305 (22.3%)	0.0086*
Geometric mean *pfs25* density	1.05 [0.76–1.46]	3.46 [2.00–5.97]	< 0.0001*

Data for both sites combined. Numbers in brackets are 95% confidence intervals; asterisk (*) indicate significant at P < 0.05; densities are determined using T-test; and differences in prevalence using the Chi-Square test

Parasite densities by qPCR did not differ between seasons (Table 1, Table 2). Across all surveys, parasite densities differed significantly between age groups (*P* < 0.001, Fig. 2). The densities peaked in children aged 5–15 years with a mean of 20.8 parasites/μL (95% confidence interval [CI95]: 12.6–34.5), and thus were 6-fold higher than in adults aged > 15 years (3.3 parasites/μL, CI95: 2.1–5.1).

### Proportion of gametocyte positive infections and gametocyte density

Across all surveys, gametocytes were detected in 119/2859 (4.2%) individuals. The population gametocyte prevalence differed significantly between sites across seasons (Table 1, wet: *P* < 0.001, dry: *P* < 0.001), and ranged from 1.4% in Homa Bay in the wet season to 11.4% in Chulaimbo in the wet season (*P* < 0.001, Table 1). The proportion of all individuals with blood stage parasites who were positive for gametocytes (the proportion of gametocyte positive infections) was significantly higher in the dry season (33.8%) than in wet season (22.3%, *P* = 0.009, Table 2, site-specific data presented in Table 1), but no difference was observed between sites (wet: *P* = 0.149, dry: *P* = 0.298).

**Fig. 3:**
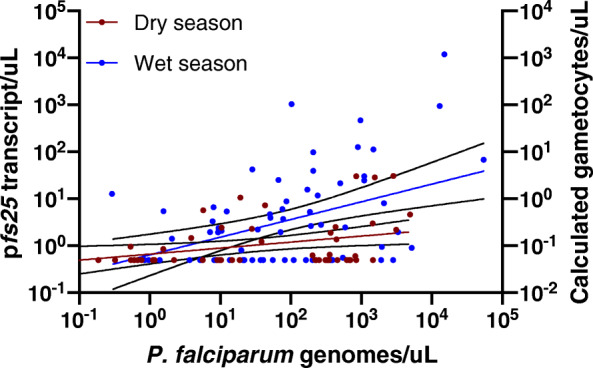
**Correlation between** ***P. falciparum*** **parasite and gametocyte densities across seasons.** Black lines show 95% confidence intervals. The left Y-axis shows the number of *pfs25* transcripts measured by RT-qPCR. The right Y-axis shows the calculated number of gametocytes, assuming 10 transcripts per gametocyte [40, 42]

The proportion of parasite and gametocyte carriers, and proportion of gametocyte positive infections was highest in school-age children aged 5–15 years across seasons and sites (Fig. 2). *pfs25* transcripts/μL differed significantly between age groups (*P* = 0.004, Fig. 2). The transcript copies/μL peaked in children aged 5–15 years with a mean of 3.6 transcripts/μL (CI95: 2.0–6.4), and thus were 3-fold higher than in adults aged > 15 years (1.1 transcripts/μL, CI95: 0.8–1.5). The correlation between *varATS* copy numbers and *pfs25* transcripts was moderate, but highly significant (R = 0.36, *P* < 0.001, Fig. 3). Likewise, the probability to detect gametocytes was correlated with parasite density. Each 10-fold increase in genome copies resulted in 3.23-fold higher odds in carrying *pfs25* transcripts*.*

### Seasonal differences in gametocyte carriage

**Fig. 4:**
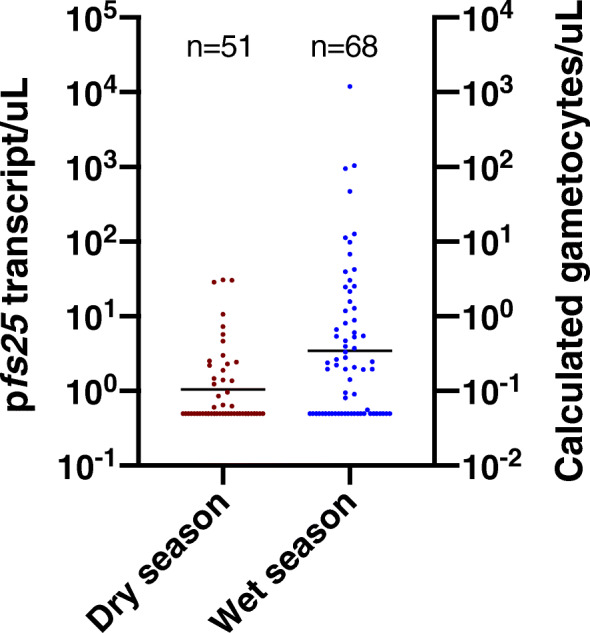
***P. falciparum*** **gametocyte densities across seasons.** The left Y-axis shows the number of *pfs25* transcripts measured by RT-qPCR. The right Y-axis shows the calculated number of gametocytes, assuming 10 transcripts per gametocyte [40, 42]. The black line shows geometric mean *pfs25* densities

Seasonal patterns in gametocyte carriage were similar in Homa Bay and Chulaimbo. Thus, results are presented for both sites combined (Table 2, site-specific data in Table 1). The proportion of gametocyte positive infections was significantly higher in the dry season (33.8%, 51/151) compared to the wet season (22.3%, 68/305, *P* = 0.009, Table 2). In contrast, mean gametocyte densities were 3-fold higher in the wet season (wet: 3.46 *pfs25* transcripts/μL (CI95: 2.0–6.0), dry: 1.05 *pfs25* transcripts/μL (CI95: 0.8–1.5), *P* < 0.001, Fig. 3, Fig. 4), even though parasite densities did not differ across seasons (wet: 8.98 *var*ATS copies/genome (CI95: 5.9–13.6), dry: 7.29 *var*ATS copies/genome (CI95: 4.2–12.7), *P* = 0.562, Table 2). The difference in *pfs25* transcript numbers between seasons remained highly significant when including log-transformed parasite densities as a predictor in multivariable analysis (Table 3). No interaction was observed between parasite density and the probability that an individual carried gametocytes, and season (*P* = 0.739).

**Table 3:** Multivariable predictors of gametocyte positivity and density

***pfs25*** positivity	aOR	***P*** value
log10 *Pf* copies	0.53	< 0.001
Wet season	−0.716	0.002
**log10** ***pfs25*** **density**	**Coefficient**	***P*** **value**
log10 *Pf* copies	0.246	< 0.001
Wet season	0.42	0.004

Only parasite density (log10 transformed) and season were found to be significant predictors for the probability that an infection was positive for *pfs25*, and for *pfs25* density. aOR = adjusted odds ratio

Pronounced variation in gametocyte carriage was observed among infections, with many medium- or high-density infections not carrying any detectable gametocytes. Among infections with a density of > 2000 *var*ATS copies/μL (corresponding to > 100 parasites/μL), in the wet season 60.0% (24/40) carried gametocytes versus 36.8% (32/87) in the dry season (*P* = 0.014). Very few individuals carried gametocytes at high densities. For example, across both surveys, only 30 individuals carried *pfs25* transcripts at densities > 5 transcripts/μL (corresponding to approximately 1 gametocyte per 2 μL of blood), 6 in the dry and 24 in the wet season. Among them, 4/6 and 19/24 were school-age children aged 5–15 years.

In multivariable analysis, only parasite density and season were found to be significantly associated with the probability that an individual was gametocyte positive (Table 3). Age group (*P* = 0.195), sex (*P* = 0.214), and site (*P* = 0.364) were not associated. Likewise, gametocyte density was only significantly associated with parasite density and season, but not site (*P* = 0.063), age group (*P* = 0.733), or sex (*P* = 0.611) (Table 3).

### Gametocyte carriage among patent and subpatent infections

A sensitivity of 100 blood stage parasites/μL (i.e. asexual parasites and gametocytes) was assumed to determine the proportion of infections that could be detected by Rapid Diagnostic Test (RDT) or light microscopy. Given this threshold, 72.1% (329/456) of all infections were subpatent across all surveys. No difference in the proportion of subpatent infections was observed between seasons (dry: 73.5% (111/151), wet: 71.5% (218/305), *P* = 0.648). 52.9% (63/119) of all infections with gametocytes detected by RT-qPCR were subpatent across all surveys, with equal proportions in the dry (52.9% (27/51)) and wet season (52.9% (36/68)). Mean *pfs25* densities were 3-fold lower in subpatent infections compared to patent infections (1.26 vs. 3.64 transcripts/μL, *P* = 0.003).

## Discussion

We observed a contrasting pattern of gametocyte carriage between the dry and the wet season in blood samples collected from 2859 afebrile individuals residing in a malaria endemic area of western Kenya. In the wet season, when most transmission is expected to occur, fewer infections harbored gametocytes. Among gametocyte-positive infections, however, gametocyte densities were higher, as was the proportion of infections harboring gametocytes at densities that could likely infect mosquitos. The higher gametocyte densities in the wet season are particularly noteworthy as parasite densities did not differ between seasons. Thus, the proportion of gametocytes among total blood stage parasites was higher in the wet season compared to the dry season. Our results imply that parasites increase their investment in gametocytes in the high transmission period to be synchronized with increased vector abundance in the rainy season.

However, the adjustment was not uniform across all infections. Less than a quarter of infections carried detectable gametocytes in the wet season. This is line with previous studies, where a majority of infections did not carry gametocytes detected by RT-qPCR [44, 45]. In some cases gametocytes might be present below the limit of detection even by RT-qPCR [46]. Yet, even among medium-to-high density infections (above 100 parasites/μL), more than half did not carry gametocytes. Given the high sensitivity of our RT-qPCR, limited detectability cannot explain this result.

Presence of *pfs25* transcripts detected by RT-qPCR does not necessarily imply infectivity. Molecular methods detect transcripts at densities at below the limit for successful mosquito infections [47], and the proportion of infections with detectable transcripts depends on the limit of detection of the molecular assay [46, 48]. Gametocyte density, and the proportion of infections with gametocytes at a density that could infect mosquitos appear to be more informative measures [23, 25]. At low gametocyte densities, mosquito infectivity increases with increase in gametocyte density. At high densities of several hundred gametocytes per uL blood, infectivity reaches saturation [25], yet very few infections in the present study were in this range.

While our quantification of *pfs25* transcripts is a good marker of infectivity at time of sample collection [23, 25, 49], it is only an indirect measure of commitment to transmission. Asexual parasite densities are expected to peak early in the infection, when mature gametocytes are not yet circulating. Likely, some of the high-density infections observed in our study were recently acquired and carried sequestered gametocytes that appeared in the blood a few days after sample collection. Among infections with above average proportions of gametocytes, asexual densities might have been higher two weeks prior when gametocyte development was initiated. Alternatively, the pattern might reflect true differences in gametocyte conversion. Few studies have measured the conversion rate directly on field isolates, and those who did found pronounced variation among *P. falciparum* isolates [32–34]. The factors underlying these differences remain poorly understood.

Our findings of higher gametocyte densities in the wet season are in line with xenodiagnostic surveys conducted from asymptomatic residents of Burkina Faso and Kilifi, Kenya. Gametocyte densities determined by molecular assays targeting *pfs25* transcripts and infectivity were substantially higher in the wet compared to the dry season [23, 24]. Similarly, the present study corroborates previous work on asymptomatic individuals in eastern Sudan [50]. These adjustments to seasonality have important implications for programs that aim to detect asymptomatic infections through population screening. In all surveys for the present study, 67–80% of infections were calculated to be subpatent (< 100 parasites/uL). In both sites and seasons, approximately half of all individuals that had gametocyte detected by RT-qPCR carried infections at densities below the limit of detection of microscopy or rapid diagnostic test. They thus would escape screening of asymptomatic individuals using field-deployable diagnostics. Gametocyte densities were 3-fold lower in subpatent individuals, yet among the 30 infections with moderate to high gametocyte densities, 11 were subpatent. Among them, 8 were sampled in the wet season. Thus, population screening would miss a much larger proportion of infections likely infective in the wet season compared to the dry season.

As opposed to Chulaimbo where parasite prevalence doubled in the wet season, in Homa Bay the prevalence did not change. The variations in seasonal parasite prevalence pattern between Chulaimbo and Homa Bay may be due to differences in species composition of local vector populations [51]. In Chulaimbo, *An. Arabiensis* forms the predominant mosquito vector species followed by *Anopheles gambiae s.s* [5]., whereas in Homa Bay *An. funestus* is the predominant mosquito vector species [37]. *An. funestus* prefers permanent bodies of water like irrigated rice fields that last beyond the wet seasons, while *An. arabiensis* prefers temporary holes and pools that dry out once the rainy season ends [52–55].

In conclusion, we have observed changes in the investment in transmission across seasons in asymptomatic *P. falciparum* infections in two sites. The increased infectivity in the wet season has important implications for control interventions. Given that it is not paralleled by increased parasite densities, screening using RDT or light microscopy in the wet season would miss an even larger proportion of the infectious reservoir than in the dry season. A small number of individuals, mostly school children, carried high gametocyte densities and likely contributed disproportionally to transmission. Targeted treatment of school children at the beginning of the wet season with gametocidal drugs such as low-dose primaquine in addition to blood-stage treatment might reduce transmission substantially [56]. Given the limited sensitivity of microscopy or RDT, this treatment should not be based on field-deployable diagnosis. Further research will be required to understand the stimuli that cause parasites to increase gametocyte density in the wet season, such as the frequency of uninfected mosquito bites [57], or they might sense physiological factors of the human body that change in response to seasonality. Surveillance systems assessing the impact of control on malaria asymptomatic reservoirs need to consider seasonal changes of gametocytemia that might differ from changes in parasitemia.

## Supplementary Information

**Additional file 1.** qPCR, RT-qPCR, and ddPCR conditions

**Additional file 2.** Database.

## Data Availability

All data generated or analyzed during this study are included in this published article and its supplementary information files.

## Abbreviations

IRS: indoor residual spraying
RT-qPCR: reverse transcriptase – quantitative polymerase chain reaction
ddPCR: droplet digital PCR
*var*ATS: *var* gene acidic terminal sequence
RNA: ribonucleic acid
DNA: deoxyribonucleic acid
EDTA: ethylenediaminetetraacetic acid
