# Supplementary material for: Increased investment in gametocytes in asymptomatic Plasmodium falciparum infections in the wet season
Source: BMC Infect Dis. 2021 Jan 9;21:44. doi: 10.1186/s12879-020-05761-6 (PMC7797145; doi:10.1186/s12879-020-05761-6)
Supplement: Supplementary file 1 — Additional file 1. qPCR, RT-qPCR, and ddPCR conditions [file 12879_2020_5761_MOESM1_ESM.docx]

**Increased Investment in Gametocytes in Asymptomatic *Plasmodium falciparum* Infections in the Wet Season**

**Additional File 1**

**Droplet digital PCR *var*ATS protocol**

| **Component** | **Final concentration** | **Volume/Reaction** |
| --- | --- | --- |
| Bio-Rad supermix for probe (No dUTP) | 1X | 11 µL |
| Probe | 10 µM | 0.55 µL |
| Primer set (Fwd+Rev) | 10 µM | 1.98 µL |
| Nuclease-free water |  | 6.47 µL |
| Template DNA |  | 2 µL |

Thermocycling conditions

95^0^C (10 min)

94^0^C (30 sec)

55^0^C (1 min) 45 cycles

98^0^C (10 min)

Primer and probe sequences

*var*ATS fwd CCCATACACAACCAAYTGGA

*var*ATS rev TTCGCACATATCTCTATGTCTATCT

*var*ATS probe 6-FAM-TRTTCCATAAATGGT-NFQ-MGB

***pfs25* RT-qPCR protocol**

| **Component** | **Final concentration** | **Volume/Reaction** |
| --- | --- | --- |
| Radiant ^TM^ 1-step Lo-Rox 2X Mix (Alkali Scientific) | 1X | 6 µL |
| 20x RTase | 1X | 0.6 µL |
| Primer set (Fw+Rv) | 10 µM | 1 µL |
| Nuclease-free water |  | 2.4 µL |
| Template RNA |  | 2 µL |

Thermocycling conditions

45^0^C (10 min)

95^0^C (2 min)

95^0^C (10 sec)

58^0^C (30 sec) 45 cycles

Primers

*pfs25* fwd CGT TTC ATA CGC TTG TAA ATG

*pfs25*_rev TTA ACA GGA TTG CTT GTA TCT AA

**Droplet digital PCR *pfs25* protocol**

Bio-Rad probe (2X supermix for probe (No dUTP)

| **Component** | **Final concentration** | **Volume/Reaction** |
| --- | --- | --- |
| Bio-Rad supermix for probe (No dUTP) | 1X | 11 µL |
| Probe | 10 µM | 0.22 µL |
| Primer set (Fwd+Rev) | 10 µM | 0.66 µL |
| Nuclease-free water |  | 8.12 µL |
| Template DNA |  | 2 µL |

Thermocycling conditions

95^0^C (10 min)

94^0^C (30 sec)

54^0^C (1 min) 45 cycles

98^0^C (10 min)

Primers and probe

*pfs25*_Probe HEX-TG TAA GAA TG TAA CTT GTG GTA ACG GT-ZEN

*pfs25*_fwd CGT TTC ATA CGC TTG TAA ATG

*pfs25*_rev TTA ACA GGA TTG CTT GTA TCT AA
